# Label‐Free Myelin Fingerprinting: An Adult Human Post‐Mortem Brain Slice Platform via Coherent Anti‐Stokes Raman Spectroscopy

**DOI:** 10.1002/advs.76471

**Published:** 2026-07-11

**Authors:** Kasra Roya‐Kouchaki, Jeroen P. Korterik, Femke Tjebbes, Geert J. Schenk, Herman L. Offerhaus, Antonio Luchicchi

**Affiliations:** ^1^ Department of Anatomy and Neurosciences Amsterdam University Medical Center VU Medical Center VU University Amsterdam The Netherlands; ^2^ Amsterdam Neuroscience Amsterdam University Medical Center Amsterdam The Netherlands; ^3^ MS Center Amsterdam Amsterdam UMC location Vrije Universiteit Amsterdam Amsterdam The Netherlands; ^4^ Optical Sciences group Faculty of Science and Technology (TNW) University of Twente Overijssel The Netherlands

**Keywords:** coherent anti‐stokes raman spectroscopy, multiple sclerosis, myelin

## Abstract

Animal models and simple cell cultures often lack sufficient overlap with human physiology to study human neurological conditions effectively; one emerging alternative is the use of human organotypic slice cultures (OSCs). This study shows, using viability studies, post‐mortem corpus callosum (CC) tissue OSCs cultured with a chemically defined serum‐free culturing medium, maintaining viability for at least 8 days in vitro (DIV). The inclusion of CC simplifies myelin‐focused research, and its potential is highlighted by visualizing molecular information within myelin by the use of label‐free Coherent anti‐Stokes Raman spectroscopy (CARS) imaging. This study delineates a window of 32 h after time of death (TOD) wherein myelin remains unaffected from base measurements. Finally, as a proof‐of‐concept, the CC tissue of a human‐specific disease characterized by myelin degeneration, multiple sclerosis (MS), reveals signs of altered myelin spectral profiles when compared to non‐MS myelin. In conclusion, this study provides characterization of the development of a human myelin model in combination with a CARS‐focused method of analysis, establishing it as a novel toolset for future research of myelin‐based neurological conditions.

## Graphical abstract

A serum‐free, air–liquid interface organotypic slice culture model preserves human post‐mortem corpus callosum tissue, successfully recovering from slicing trauma to reflect the donor's underlying disease state. Pairing label‐free Coherent anti‐Stokes Raman scattering (CARS) microscopy with k‐means clustering enables objective, high‐resolution structural mapping to uncover subtle myelin features otherwise invisible to conventional imaging techniques.

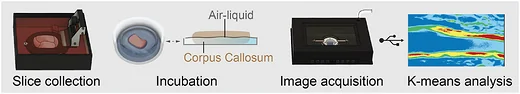

## 1 Introduction

The use of animal models and simple cell cultures has expanded our understanding of neurological conditions, like multiple sclerosis (MS) and neurodegenerative diseases, but they do not capture the intricate environment in which these disorders develop in humans; and in the vast majority of cases, animal studies do not lead to successful clinical translation in humans [1]. A recent paradigm shift has been the consequence; the Food and Drug Administration (FDA) and the European Medicines Agency (EMA) updated their regulatory framework and established clear pathways toward validation for human‐data‐driven New Approach Methodologies (NAMs) to replace animal data in preclinical drug evaluation [2, 3]. Acute‐ and organotypic slice cultures (OSCs) are an example of NAMs and have provided avenues to maintain complex human‐specific cell‐to‐cell interplay, non‐conserved biological processes, and much of the original architecture of the brain ex vivo. In comparison to animal studies, using human brain tissue combined with the administration of putatively therapeutic compounds could provide improved preclinical validations, safer transitions toward human clinical trials, and altogether reduce the reliance on animal experiments.

One major breakthrough in OSC development was the placement of slices on semi‐permeable membranes at the air‐liquid interface [4], which provided a simplified follow‐up to the preceding roller‐tube technique [5, 6]. The majority of these methods have been developed and optimized in rodents [4, 7, 8, 9, 10, 11, 12, 13]. Most commonly, OSCs using human brain tissue rely on resected tissue from donors requiring brain surgery [14, 15, 16, 17, 18, 19, 20], and to a lesser extent, on fetal‐derived tissue [21, 22, 23]. Post‐mortem tissue is an alternative source material that covers a wider range of pathological and non‐pathological conditions, and brain regions normally not accessible during surgery or not fully developed in fetuses. Initial research on post‐mortem OSCs [24, 25, 26, 27, 28] has recently been adapted to the aforementioned air‐liquid interface method [29, 30, 31, 32], simplifying its use and allowing for wider adaptation.

Our present study aims to (1) create an OSC method containing corpus callosum (CC) tissue rich in highly ordered white matter (WM) fibres, (2) to simplify and advance myelin‐study possibilities, (3) develop a new chemically defined cerebrospinal fluid (CSF)‐like culturing medium to reduce variability and increase biological relevance, and (4) to find a culture‐window where tissue remains viable and myelin (largely) unaffected. For data acquisition and analysis, respectively, we utilised anti‐Stokes Raman spectroscopy.

(CARS) in combination with *k*‐means clustering to detect minute myelin alterations. CARS is a selective label‐free imaging technique for chemical composition based on vibrational contrast [33, 34, 35]. It allows for sub‐micron spatial resolution, with little perturbation to the original cellular environment, while providing information about the chemical bonds within a sample. Prior use of *k*‐means clustering using CARS imaging of biological samples [36, 37] demonstrated data‐driven distinctions in areas within axonal myelin. Finally, we included MS tissue, expecting our method to be sensitive to the differences between MS and non‐MS myelin, thereby providing a proof‐of‐principle for future research. MS tissue stands as a model of a chronic neurodegenerative and neuroinflammatory disease of the CNS, characterized by a progressive loss of myelin [38]. Observations on human MS post‐mortem normal‐appearing WM [39, 40] have shown the presence of local alterations in the form of so‐called myelin blisters; subtle swelling‐like forms of myelinic detachment from axonal processes able to alter the axon‐myelinic communication [41]. Since comparative dynamic systems allowing for the study of these minute myelin alterations were required but lacking, this current study provides a novel set of tools capable of filling that gap.

In this study, we show that OSCs from human CC maintain stable myelin morphology for at least 32 h after time of death (TOD) and cell viability for 200 h after TOD, or a lower‐bound approximation of 1 and 8 days in vitro (DIV), respectively. CARS analysis also reveals that this method is sensitive to subtle changes in myelin biochemical composition during long‐term culturing, a feature not ascertainable by live‐cell imaging using conventional fluorescent dyes, e.g., FluoroMyelin. Finally, we provide evidence for an overall reduction in mean CH_2_ peak intensity and an altered distribution of CH_2_ peaks located at sites of swollen myelin, when comparing MS and non‐MS CC‐myelin prior to culturing, suggesting that the emergence of morphological alterations in MS, like myelin swellings, can be coupled to biochemical alterations at the level of myelin.

## 2 Materials and Methods

### 2.1 Donor Material and Sample Preparation

Freshly excised coronal or sagittal brain blocks encompassing a portion of the anterior (post‐genu) trunk of WM CC, with attached grey matter (GM) cingulate gyrus for orientation purposes, were provided by the Netherlands Brain Bank (NBB) and Normal Aging Brain Collection Amsterdam (NABCA). In addition, visible lesions were avoided, and only donors with a post‐mortem delay (PMD) of less than 8 h were included. In total, tissue from 9 MS cases and 7 non‐MS controls was used for the study. The mean age of death of the donors was ∼62 ± 8 for MS and ∼82 ± 10 for controls. The mean PMD was ∼6 h and was calculated from the moment the donor departed to the moment of storage of the tissue in the medium. Tissue preparation and handling took on average 2 h, with a range of 30 min to 3.5 h. Other donor‐specific details are present in Table 1.

**TABLE 1:** Demographic and clinical data for post‐mortem cases. PMD, Post‐mortem delay; MS, Multiple Sclerosis; EdcrD, Endocrine disease; CVD, Cardiovascular disease, SjD; Sjögren's disease; PSP, Progressive supranuclear palsy; LBD, Lewy‐body disease; AD, Alzheimer disease; NNC, confirmed non‐neurological control; MTS, 3‐(4,5‐dimethylthiazol‐2‐yl)‐5‐(3‐carboxymethoxyphenyl)‐2‐(4‐sulfophenyl)‐2H‐tetrazolium inner salt; LDH, Lactate dehydrogenase; L/D, Live/dead; CARS, Coherent anti‐Stokes Raman spectroscopy; GS, Glutamine Synthetase; GFAP, glial fibrillary acidic protein; MHCII, Major histocompatibility complex II; IBA1, Ionized calcium‐binding adaptor molecule 1.

Donor ID	Sex	Age at Death	Diagnosis	Cause of Death	∼PMD (h)	Used for
MS1	F	71	MS	Euthanasia	7.5	LDH MTS L/D CARS
MS2	M	54	MS	MS	6.5	MTS DAPI
MS3	F	76	MS	Atrial fibrillation ‐medication halted	6	LDH
MS4	F	67	MS	MS	7	LDH GFAP MHCII
MS5	F	69	MS	Metastatic Collon cancer‐palliative care	5.5	LDH
MS6	F	54	MS	Euthanasia	4.5	LDH DAPI GFAP MHCII
MS7	F	73	MS	Deceased in sleep	7	LDH DAPI GFAP MHCII
MS8	F	50	MS	Euthanasia	4.5	LDH DAPI GFAP MHCII
MS9	M	51	MS	MS	8	MTS
NM1	F	91	Non‐MS (EdcrD& CVD)	Cardiac arrest	6	LDH DAPI GFAP MHCII
NM2	F	94	Non‐MS (SjD & CVD)	Pneumonia	3.5	LDH DAPI GFAP MHCII FLML CARS
NM3	M	76	Non‐MS	Prostate cancer—Euthanasia	7	LDH DAPI GFAP MHCII
NM4	M	90	Non‐MS (PSP)	Euthanasia	5	DAPI GFAP GS IBA1 MHCII FLML CARS
NM5	M	80	Non‐MS (LBD—AD)	Pneumonia	6	LDH DAPI GS IBA1 GFAP MHCII
NM6 NM7	F M	79 63	Non‐MS Non‐MS (NNC)	Euthanasia Euthanasia	4 6	L/D DAPI GS GFAP IBA1 MHCII

For transport, brain blocks were stored in non‐supplemented room temperature (RT) (or ambient temperature) Hibernate‐A medium (A1247501; Gibco, U.S.A.) immediately after excision and during any extended situation wherein the oxygen and temperature could not be controlled. For imaging and culturing, the tissue was maintained in culture medium at 35 or 37°C, in miniature and large incubators, respectively.

For all experiments with human tissue, we adhered to the Netherlands Code of Conduct for Research Integrity and the Declaration of Helsinki, with protocol approval by the Medical Ethical Committee of the Amsterdam UMC. The entire study was performed in strict compliance with ethical requirements of the Amsterdam University Medical Centers, VU Medical Center, Amsterdam. Informed consent was requested by the NBB and NABCA and granted by the donors.

### 2.2 Tissue Slices

Post‐mortem CC tissue blocks were cut, in RT hibernate‐A medium, in 200 µm and 450 µm‐thick slices with a vibrating blade microtome / vibratome (VT1000S; Leica Biosystems; Germany) preserving the parallel orientation of axonal tracts (Figure 1). Additionally, thicker slices of ∼1400 and ∼2000 µm were cut using a custom‐made slice matrix and a commercial option (TM‐2000; ASI Instruments; USA), respectively. All slices were subsequently transferred to either FluoroDishes (FD35; World Precision Instruments; U.S.A) filled with 1–2 mL media, or standing cell culture inserts (PICM0RG50; Millipore; Germany) on 6‐well plates (140675; Nunc A/S, Denmark) with 1 mL media, for CARS imaging and slice culturing, respectively. Slices were transferred from the Hibernate‐A storage medium using small spatulas, with excess fluid gently drained prior to placement onto the culture inserts. This approach was selected to minimize direct mechanical handling and preserve the structural integrity of the delicate post‐mortem tissue, while ensuring a rapid transition to the definitive culture environment. Slices were maintained at the air‐liquid interface as described by Stoppini et al. [4]. To limit physical drift of the tissue during imaging, we used a custom‐made tissue holder to keep them in place (Figure 1 and more extensively described in Figure S1). The CARS slices were maintained, while imaged, in a miniature incubator (TC‐MWP & TC‐MWPL; Biosciencetools; U.S.A.) at 35°C supplied with carbogen gas with 5% CO_2_ and 95% O_2_, whereas the cultures were maintained in humidified incubators at 37°C with 5% CO_2_ and 95% O_2_. After various incubation times, we imaged live slices by either CARS or confocal imaging, or froze the slices using liquid nitrogen. Frozen tissues were stored at ‐80°C.

**FIGURE 1:**
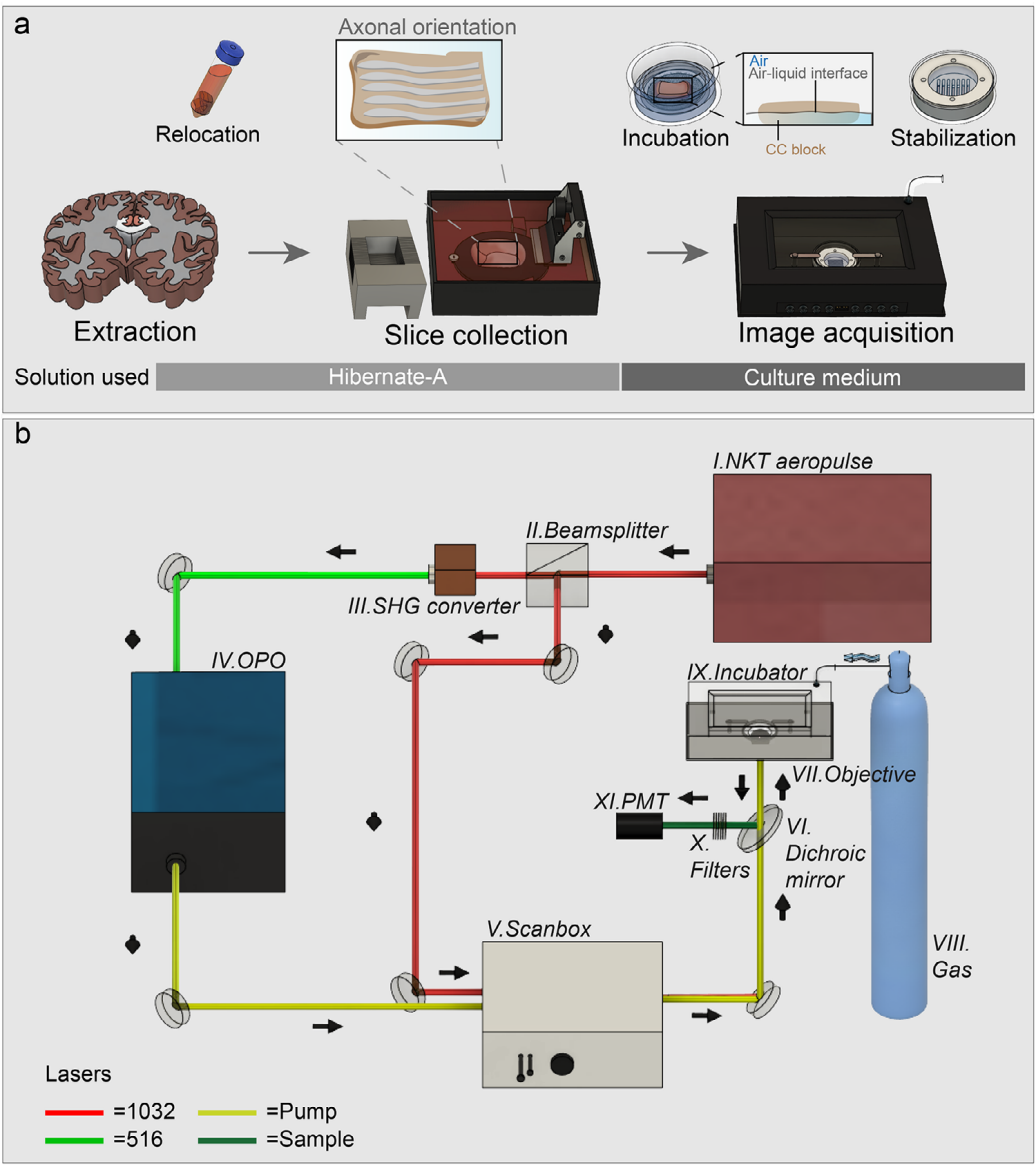
Overview of tissue extraction, sectioning, and CARS microscopy setup. (a) During autopsy, the anterior corpus callosum trunk (post‐genu), including the attached cingulate gyrus for orientation, was dissected and stored in room‐temperature Hibernate‐A medium. Vibratome sectioning produced 200 µm and 450 µm thick slices, cutting in an orientation intended to maximise parallel axons, in room‐temperature Hibernate‐A medium; a slice matrix was used to create ∼1.4 and ∼2 mm thick slices. Slices were cultured at the air‐liquid interface in 6‐well plates using cell culture inserts with culture medium. For CARS‐imaging, slices were transferred to FluoroDishes containing culture medium and secured using a custom holder. (a‐b) The sample was maintained in a miniature incubator (IX) at 35°C under a carbogen‐gas (VIII) atmosphere (5% CO2, 95% O2). (b) An NKT AeroPulse laser (I) generated 1032 nm picosecond pulses, i.e., Stokes beam (red). A beam splitter (II) directed the Stokes beam to the scan box (V) and to a second harmonic generation (SHG) converter (III). The SHG converter doubled the Stokes beam frequency to 516 nm (light green). The tunable pump beam (yellow) from the optical parametric oscillator (OPO) (IV) was combined with the Stokes beam. The combined pump and Stokes beams were aligned, directed toward the objective lens (VII), and focused onto the sample. The backscattered CARS signal was reflected by a dichroic mirror (VII), filtered by a filter set (X), and detected by a photomultiplier tube (PMT) (XI). SHG, second harmonic generation; OPO, optical parametric oscillator; PMT, photomultiplier tube.

### 2.3 Media Composition

For acute experiments, we adapted a version of artificial Cerebrospinal fluid (aCSF) [15] containing; 125 mM NaCl (31434, Riedel‐de‐Haën, U.S.A.), 15 mM KCl (4936, Merck, Germany), 1.25 mM NaH_2_PO_4_ (106346, Merck, Germany), 24 mM NaHCO_3_ (6329, Merck, Germany), 12.5 mM D‐Glucose (G8270, Sigma‐Aldrich, U.S.A.), 1.6 mM CaCl_2_·2H_2_O (12064; Riedel‐de‐Haën, U.S.), and 2 mM MgSO_4_·7H_2_O (5886, Merck, Germany). Later on, to improve long‐term viability while limiting variability, we created our enhanced chemically defined‐slice culture media (eSCM) consisting of Minimum Essential Medium (MEM) containing no glutamine and no phenol red (51200038; Gibco; U.S.A.). We adjusted the base media with Milli‐Q solutions containing NaCL (AM9760G; Invitrogen; U.S.), MgSO_4_·7H_2_O (5886; Merck; Germany), D‐Glucose (G8769; Sigma‐Aldrich, U.S.A), and GlutaMAX‐I supplement 100x (35050‐038; Invitrogen; U.S.A.). Highlighted, the final combined concentrations were; 120 mM NaCL, 5.28 mM KCl, 1 mM NaH_2_PO_4_, 25.92 mM NaHCO_3_, 5.5 mM D‐Glucose, 1.8 mM CaCl_2_, and 1 mM MgSO_4_. Due to the slight increase in volume, MEM media amino acids and vitamins were reduced to 99% of their initial values available from the manufacturer. The culture media were then filter sterilised with 0.22 µm filters (76479‐044; VWR International, U.S.A) and stored at 4°C. For the cultured slices, we collected and refreshed culture media every 16 h for the first 48 h. After the first 48 h of total time in an incubator, 1% v/v Penicillin‐streptomycin (15140‐122; Invitrogen, U.S.A.) was added, and culture media were collected and refreshed every 48 h until final collection at DIV7 or DIV8.

### 2.4 Calcein AM and 7‐AAD Assay

Live cell imaging was performed on specific tissues that had spent ∼7.5 h in Hibernate A to simulate extraneous situations where tissue handling and preparation might be prolonged, in order to represent a worst‐case scenario. After different amounts of time spent in an incubator some slices were incubated for 40 min in a humidified incubator at 37°C with 5% CO_2_ and 95% O_2_ in presence of 0.5 µM 7‐aminoactinomycin‐D (7‐AAD) (00‐6993‐50; Invitrogen, U.S.A.) and 5 µM Calcein acetoxymethyl (Calcein AM) (PF00007; Proteintech, U.S.A.). Calcein AM and 7‐AAD were dissolved in aCSF or culture media, according to experimental needs. Subsequently, slices were washed out with aCSF or culture media (2 × 10 min). Finally, the tissue was placed back in the incubator and imaged within 24 h. Fluorescence imaging was carried out using either a Nikon‐A1 (Nikon Corporation, Japan), a Leica TCS SP8 (Leica Biosystems, Germany), or a Leica LSM‐880 (Leica Biosystems, Germany) confocal microscope. The percentage of live, damaged, and dead cells was determined using ImageJ (National Institutes of Health, U.S.A). Post‐processing was handled as described in the Figure S2.

### 2.5 FluoroMyelin

For fluorescence imaging of myelin, live slices were incubated with FluoroMyelin Green (1:150, F34651, Invitrogen, U.S.A.) dissolved in our eSCM for 30 min. This was followed by 3 × 10 min washes using adjusted custom media composition, after which slices were imaged using a Leica TCS SP8 (Leica Biosystems, Germany). All incubations were done in controlled humidified 37°C environments with 5% CO_2_ and 95% O_2_.

### 2.6 Frozen‐Tissue Histology

To allow for immunohistochemistry (IHC) for the assessment of tissue immune environment and cell count, frozen tissues were further sliced at 10 µm using a cryostat (CryoStar NX50; Thermo Scientific; U.S.A.), placed on glass slides (SuperFrost Plus; Thermo Fisher; U.S.A.), warmed for 15–30 min on a slide warmer, and stored at ‐80°C. The IHC protocol started with the fixation of slides in cold aceton (010306, biosolve, France) for 10 min, dried shortly with compressed air, fixated again for 15 min and again dried with compressed air before washing in Tris‐Buffered Saline (TBS). All steps were followed by 3 × 5 min washes in TBS unless otherwise specified. Heat‐induced antigen retrieval was performed by cooking slides for 12.5 min in 10 mM citrate buffer (pH 6.0) using a steam‐cooker and cooling to RT before subsequent steps. A 1 h block was performed using 3% normal donkey serum (NDS) and TBS + 0.1% Triton X‐100 (8603, Merck, Germany); this was followed directly by 30 min RT + overnight incubation at 4°C with primary antibodies dissolved in TBS + 3% NDS. The following day, blocking of endogenous peroxidases was performed by incubating in TBS+ 0.3% H_2_O_2_ for 15 min. The initial secondary antibody incubation consisted of 1.5 h of incubation at RT with Alexa 488 donkey Anti‐chicken (1:400, 703‐545‐155, Jackson ImmunoResearch Laboratories Inc, U.S.A.). Slides were incubated with 2 drops of anti‐rabbit/mouse horseradish peroxidase (poly‐HRP)‐conjugated immunoglobulins (Dako REAL EnVision HRP Mouse (ENV), K4001, Agilent technologies, U.S.A) for 30 min, and, after subsequent washing, slides were incubated with Alexa 555 Tyramide conjugates (1:100, A‐K079, Invitrogen, U.S.A.) + 0.0015% H_2_O_2_ was incubated for 20 min. Finally slides were counterstained with DAPI (1:1000, D9542, Sigma Aldrich, U.S.A.), washed 1x in TBS, and mounted with glass coverslips using a combination of Mowiol (Sigma–Aldrich) plus anti‐fading agent 1,4‐diazabicyclo[2.2.2]octane (DABCO).

Primary antibodies used were mouse anti‐Human MHCII (1:400, Clone LN3, MA5‐11966, Thermo Scientific; U.S.A.) and chicken anti‐human GFAP (1:500, AB5541, Sigma–Aldrich, U.S.A). Follow‐up experiments utilized this baseline protocol, incorporating rabbit anti‐IBA1 (1:100, 019‐19741, Wako, Japan) and mouse anti‐GS (1:100, LS‐B2579, LSBio, USA) as primary antibodies alongside the GFAP antibody. The next day, IBA1 was detected by pooling donkey anti‐rabbit Alexa 594 (1:400, A32754, Thermo Scientific, USA) into the initial 1.5‐hour secondary step, while the mouse anti‐GS primary was subsequently visualized via the EnVision‐tyramide workflow. For NM7 samples, the post‐tyramide wash was followed by a 1.5‐hour RT incubation with Alexa 647‐conjugated anti‐human MHCII (1:3, clone LN3, 327012, BioLegend, USA).

### 2.7 LDH‐Cytotoxicity Assay

Tissue culture media was collected at each scheduled sampling interval and stored at 4°C, after which the wells were completely replenished with fresh culture medium. The LDH cytotoxicity assay (C20301; Invitrogen, U.S.A.) was performed protected from light at RT in a 96‐well plate (167008; Nunc A/S, Denmark) format where 50 µL tissue culture medium was mixed with 50 µL reaction mixture. The colorimetric conversion was stopped after 30 min with 50 µL stop solution, and the absorbance was read at 490 and 680 nm. The absorbance at 490–680 minus the plate blank determined the final LDH value per sample. To obtain a percentage of cytotoxicity, a thickness‐matched slice was treated with 5% Triton X‐100 for 16 h, and its LDH value served as a maximum (100%) cytotoxicity control. To correct for variations in total slice volume, all values were further normalized to individual slice surface areas, which were quantified using ImageJ software based on the known physical dimensions of the well‐plates or FluoroDishes.

### 2.8 MTS Assay

Once the tissue had spent certain amounts of time in the incubator tissue was collected and the 3‐(4,5‐dimethylthiazol‐2‐yl)‐5‐(3‐carboxymethoxyphenyl)‐2‐(4‐sulfophenyl)‐2H‐tetrazolium, inner salt (MTS) assay (G3582; Promega, U.S.A.) was performed in a 6‐wells format. To 1 mL culture medium, we added 200 µL MTS assay mixture and subsequently incubated slices for 3 h in an incubator at 37°C with 5% CO_2_ and 95% O_2_. Media was transferred to a 96‐well format, and absorbance was read at 490 nm. The absorbance at 490 nm minus the plate blank determines the MTS value per sample. To obtain a percentage of viability, another MTS assay was initiated within an hour of receiving tissue after autopsy without additional incubation. The subsequent MTS value was used as a normalisation value for 100% viable tissue. A further normalisation based on size was performed similar to that of the LDH assay described in the previous section.

### 2.9 Coherent Anti‐Stokes Raman Scattering

A representation of the laser setup is shown in Figure 1. The setup was powered by a pulsed laser (aeroPulse PS, NKT Photonics, Denmark, 5 picosecond pulse width) operating at 1032 nm providing the ‘Stokes’ beam. Doubled 516 nm pulses from the Stokes laser drive a tuneable optical parametric oscillator (OPO, Levante Emerald PS, APE, Germany). The tuneable signal beam (called ‘Pump’) from the OPO was combined with the Stokes beam to allow for the excitation of a wide range of vibrational energy levels. The beam was scanned over the sample with a scan speed of 5.92 s/scan, and the subsequent epi‐detected CARS (E‐CARS) signal was collected by the photomultiplier tube (PMT) (Hamamatsu R3896 PMT, Japan). Pictures were acquired using an inverted microscope (Olympus IX71 with FV300 galvoscanner, Olympus, Japan, N.A. objective 1.20) at a 512 × 512‐pixel resolution. A combination of (customized in‐house) LabView (National Instruments, U.S.A.) and FluoView (Olympus, Japan) controlled the setup and scanning.

### 2.10 Spatial Sampling and Depth Selection Rationale

To account for depth‐dependent variations and surface artifacts induced by mechanical slicing, tissue evaluation was partitioned by layer according to assay sensitivity. Bulk biochemical viability (LDH and MTS) was measured across the entire slice volume to capture aggregate metabolic activity. Live‐cell surface‐layer imaging (Calcein‐AM/7‐AAD) was utilized at the tissue boundaries to establish an upper‐limit quality control checkpoint for mechanical trauma. Conversely, structural and target‐specific evaluations were localized to the unperturbed internal core; immunohistochemical quantification was restricted to the internal layers to ensure uniform antibody penetration, and long‐term CARS imaging was deliberately transitioned to the core by DIV 8 to avoid handling‐induced surface myelin degradation.

### 2.11 Coherent Anti‐Stokes Raman Scattering Image Analysis

While our custom‐made holder reduced the amount of drift, higher levels of magnification still experienced pixel‐level drift. To compensate for this, we used the fast4dreg [42, 43] ImageJ macro and applied it to our images, or adjusted manually if necessary. The resulting hyperspectral data cubes were processed and analysed using MATLAB 2023a (Mathworks, U.S.A) software. Because of power fluctuation throughout different Raman wavenumbers (2800, 2850, 2900, and 2950 cm^−1^), first a normalisation took place using the registered power‐values per wavenumber and the signal was converted to grey‐values. Subsequently, a k‐means clustering segmentation was performed with 5 clusters with a maximum of 100 iterations. Clusters were coloured and represented in an image alongside the mean intensity per Raman wavenumber. MATLAB code is provided in the supplemental information.

### 2.12 Statistical Analysis

We performed statistical analysis using Prism version 8.0 (GraphPad Software, San Diego, CA). All statistics performed on viability data were done using Sidak's multiple comparisons one‐way analysis of variance (Anova). For MHCII and GFAP data, we performed Tukey's multiple comparisons one‐way Anova, except for GFAP branch count and length, which were two‐way Anova's.

## 3 Results and Discussion

### 3.1 OSCs are Viable up to 8 Days In Vitro

After obtaining the post‐mortem tissue (Figure 1a), we assessed the viability of our OSCs employing different assays, for different incubation times and multiple slice thicknesses. Here, cohorts were pooled to reflect the system's overall broad donor viability, rather than evaluate the effects of individual pathological susceptibility. An LDH assay, showing release of cytosolic LDH by membrane‐compromised cells, reported a modest 1.5 fold increase in the LDH release in the medium after 34 h of incubation (Figure 2A; 16 h incubation: 16.47% ± 1.74; n = 17, 34 h incubation: 24.57% ± 2.28, n = 8, data normalized to 100% lysis control; Sidak's multiple comparisons one‐way Anova, p < 0.05). This indicates that the majority of damage likely occurs within the initial 16 h post‐slicing, allegedly instigated by the slicing procedure itself, further supported by prolonged LDH release on 0.45 mm and 1.4 mm‐thick slices that showed an LDH stabilization after DIV1. In contrast, 2 mm‐thick slices exhibited a delayed stabilization in LDH levels, with a prolonged period of elevated LDH release. LDH values were not significantly predicted by age or PMD (Multiple linear regression with interaction, 16 h: n = 11, 34 h: n = 6; all model and predictor p > 0.05).

**FIGURE 2:**
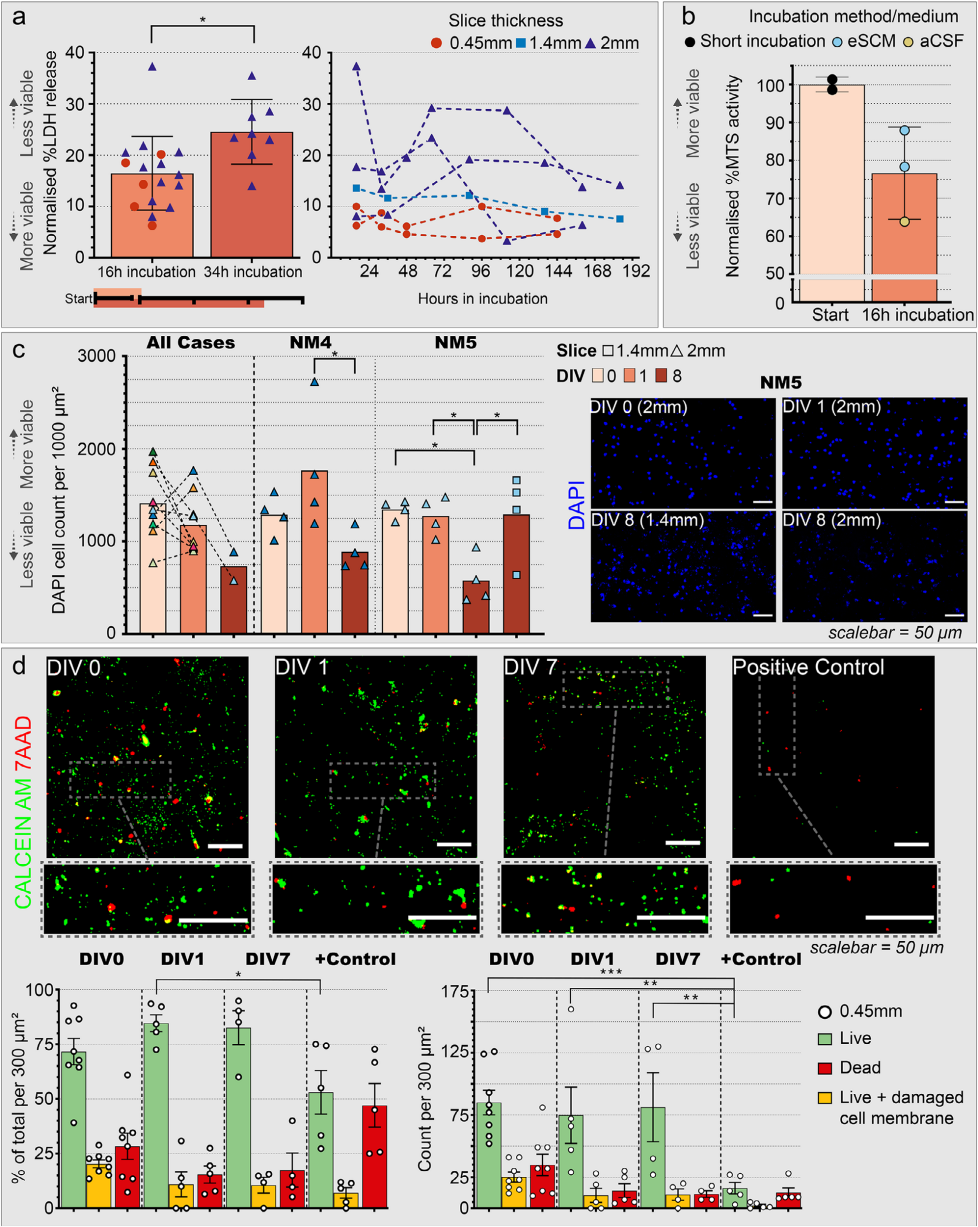
LDH, MTS, DAPI counts, and fluorescent live‐cell imaging OSC‐viability. For all graph symbols: circles, squares, and triangles denote slices 0.45, 1.4, and 2 mm thick, respectively. Error bars show standard deviation. Scale bars: 50 µm. (a) On the left graph, normalised percentage of LDH release over initial 16 h (n = 17) and 34 h (n = 8) of incubation. On the right graph, at 7‐ 8 DIV of incubation, each symbol denotes a moment of medium change and collection (n = 6) (n = slice, over unspecified number of donors). (b) All slices 0.45 mm. Normalised percentage of MTS activity on slices collected with minimal time after autopsy and slices collected after 16 h of incubation. Black, blue, and yellow circles denote Hibernate‐A only (n = 2), eSCM (n = 2), and aCSF (n = 1) medium, respectively (n = slices, over unspecified number of donors). (c) All slices 2 mm thick, except NM4 with a 1.4 mm slice. DAPI cell count over an area of 1000µm2. All cases graph group displays counts on DIV0 (n = 9), DIV1 (n = 9), DIV8 (n = 2) (n = donor‐mean, each data‐point an average of 1–2 slices, with 2–4 slice‐regions per slice). Matching colours and connecting lines denote data from the same donor. NM4 and NM5 display slice means (n = region within singular slice). On the right, confocal images of DAPI‐stained slices from NM4 over 0‐1‐8 DIV with differing slice thicknesses. (d) All slices 0.45 mm. On the top, confocal images of dual (Calcein AM & 7‐AAD) stained slices over 0‐1‐7 DIV, along with a 2% TX100 positive (high cell‐death) control collected on DIV1. Below, on the left, displaying the percentage of the whole per category, and, on the right, cell counts per category, both over ∼300 µm2 (n = slice region). In both the images and the graphs: green, red, and yellow denote live, dead, and live with a damaged cell membrane, respectively. Sidak's multiple comparisons one‐way Anova was performed for all statistical tests (^*^ *p* < 0.05, ^**^ *p* < 0.005, ^***^ *p* < 0.0005). LDH, Lactate Dehydrogenase; MTS, 3‐(4,5‐dimethylthiazol‐2‐yl)‐5‐(3‐carboxymethoxyphenyl)‐2‐(4‐sulfophenyl)‐2H‐tetrazolium; eCSM, enhanced chemically defined‐slice culture media; aCSF, Artificial cerebrospinal fluid; DIV, days in vitro; Calcein AM, calcein acetoxymethyl; 7AAD, 7‐amino‐actinomycine D.

OSCs from human CC also showed metabolic activity after 16 h of incubation, as shown by MTS assay measurements. When normalized to two freshly excised and briefly Hibernate‐A‐protected slices from one donor, OSCs showed ∼76.61% MTS activity after 16 h, confirming the slice viability indicated by the LDH assay. Interestingly, slices incubated in eSCM presented a higher metabolic activity than slices incubated in a more standard artificial CSF formulation (Figure 2B). As such, we proceeded further with our study employing eSCM only.

Nuclear density remained stable through culturing conditions on average between all cases (DIV0, n = 9; DIV1, n = 9; DIV8, n = 2; Sidak's multiple comparisons one‐way Anova, p > 0.05). NM4 and NM5 were viewed separately to show inner‐slice variability and change through DIVs; indeed a marked difference was present when we looked at in‐slice differences for these two cases (NM4+NM5), with nuclear density dropping at DIV8 for 2 mm, but not for 1.4 mm‐thick slices (Figure 2C, Sidak's multiple comparisons one‐way Anova, p < 0.05). Finally, employing a Calcein acetoxymethyl (Calcein AM) and 7‐aminoactinomycin‐D (7‐AAD) assay, we investigated the proportion of live, dead, and live but damaged cells throughout culturing. We found a stable proportion of viable cells across different DIVs, and significantly lower viable cell counts from the measurements after application of Triton X‐100 (32 h) as a positive control (Sidak's multiple comparisons one‐way Anova, *p* < 0.005 for DIV1 and DIV7, and p < 0.0005 for DIV0). Collectively, these results support that our custom eSCM sustains slice viability for up to 7–8 DIV, offering a reliable platform for extended slice culturing.

### 3.2 Pre‐Existing Disease Baseline Dictates Microglial MHC‐II Trauma Responses While Astrocytes Maintain Homeostatic Stability

We next assessed whether slicing or culturing conditions induced damage responses that might be unrelated to the disease state by monitoring microglia activation and astrogliosis. For this purpose, we evaluated the presence of major histocompatibility complex class II (MHC‐II), ionized calcium‐binding adapter molecule 1 (IBA1), glutamine synthetase (GS), and glial fibrillary acidic protein (GFAP), which are markers for antigen‐presenting cells (APCs), pan‐microglia, pan‐astrocyte, and reactive astrocytes, respectively. The previously mentioned NM4 and NM5, and newly added NM7, are displayed separately to show variability between disease states, alongside an average of all 3 cases, displaying overall method trend.

At DIV 0, MHC‐II staining showed predominantly amoeboid morphology, indicative of phagocytic activity (Figure 3a). A more heterogeneous morphological profile was visible at DIV 1, including more inactive ramified, hyper‐ramified (suggestive of cellular stress) [44, 45, 46, 47], and ball‐and‐chain microglia, the latter potentially representing the phagocytosis of small amounts of material [45]. By DIV8, MHC‐II cells presented with a mix of bushy and transitional morphologies, hinting at intermediate activation, and the reappearance of amoeboid forms, possibly related to the extended culturing time. The overall morphological profiles indicate a functional APC population without evidence of sustained inflammation.

**FIGURE 3:**
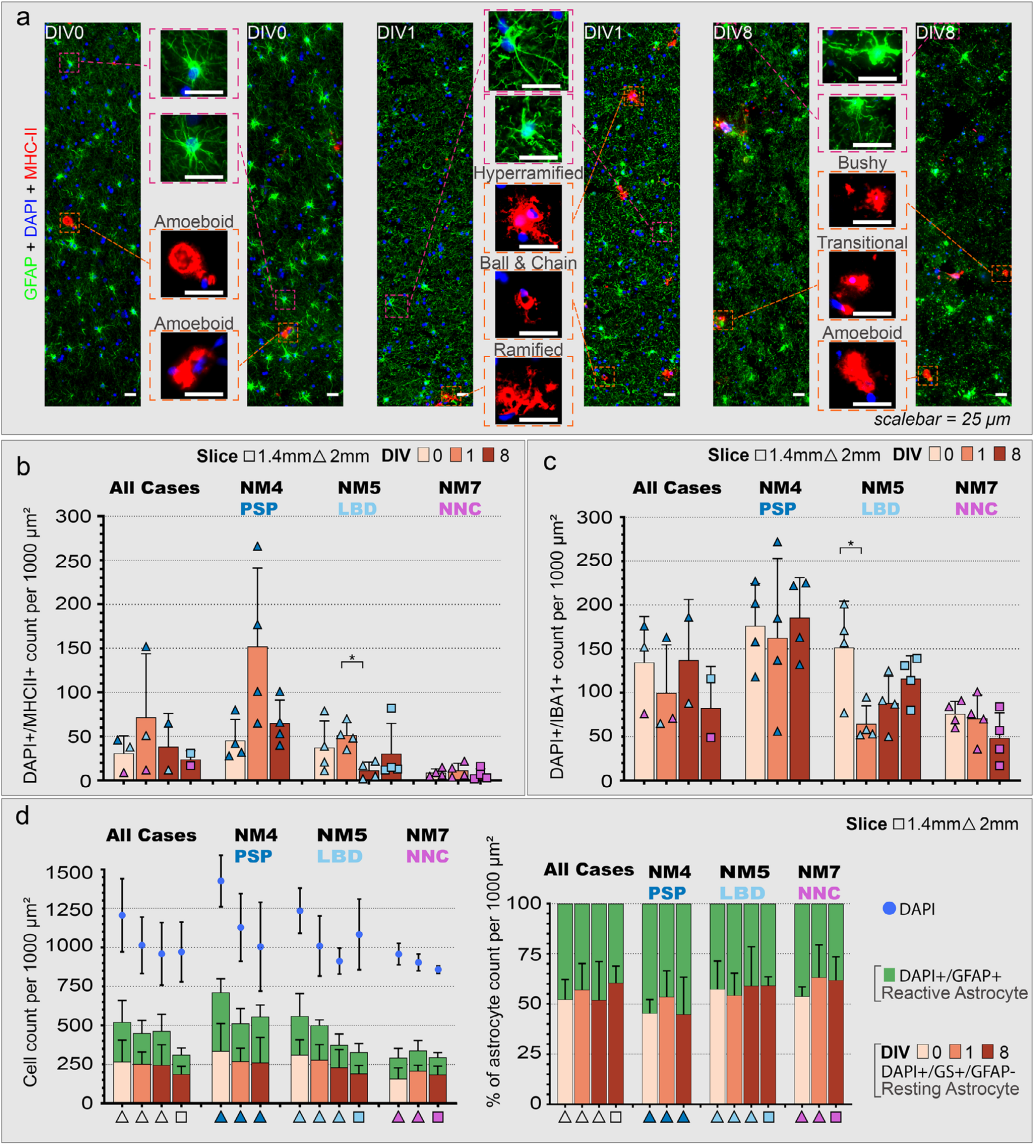
IHC‐staining of MHCII, IBA1, GS, and GFAP in OSCs. For all graph symbols: squares and triangles denote slices 1.4 and 2 mm thick, respectively. Error bars show standard deviation. Scale bars: 25 µm. (a) Confocal images of triple(GFAP, DAPI & MHCII)‐stained slices over 0‐1‐8 DIV. green GFAP, blue DAPI, and red MHCII display astrocytes, cell‐nuclei, and antibody presenting cells, respectively (b‐d) “All Cases”‐graph groups display counts across DIVs (n = 3) (n = donor‐mean, each data‐point is an average of 4 slice‐regions within a singular slice). Matching colours denote data from the same donor. NM4, NM5 and NM7 display slice means across DIVs (n = 4) (n = region within a singular slice). (b) DAPI+/MHCII+ count over an area of 1000µm^2^. (c) DAPI+/IBA1+ count over an area of 1000µm^2^. Tukey's multiple comparisons one‐way Anova was performed for all statistical tests (^*^ *p* < 0.05, ^**^ *p* < 0.005, ^***^ *p* < 0.0005). GFAP, glial fibrillary acidic protein; MHCII, major histocompatibility complex class II; DIV, days in vitro; IBA1, Ionized calcium‐binding adaptor molecule 1; GS, Glutamine Synthetase; PSP, Progressive supranuclear palsy; LBD, Lewy‐body disease; NNC, confirmed non‐neurological control.

We observed a non‐significant trend toward a transient increase of MHC‐II cell density (DAPI+/GFAP+) (Figure 3b) at DIV 1 compared to DIV0, which we suspect represents an acute inflammatory response to the tissue‐slicing and handling procedure. Following this initial peak, MHC‐II counts declined by DIV8, reaching statistical significance within the 2 mm slice of the LBD case (NM5) (Tukey's multiple comparisons one‐way Anova, p < 0.05), suggesting that OSCs successfully resolve acute activation once the mechanical slicing trigger subsides rather than persisting in a chronic inflammatory state. Notably, the NNC (NM7) maintained low MHC‐II expression throughout the culture period.

To assess the total microglial pool independent of activation status, pan‐microglial IBA1 staining was performed (Figure 3c). Mirroring MHC‐II results, the non‐neurological control (NM7) presented with a low‐density microglial population that seemed stable across the investigated DIVs. The PSP case (NM4) exhibited similar microglial population stability and resilience to slicing as the control. Conversely, corresponding to the mechanical trauma of slicing, the 2‐mm slices of the LBD case (NM5) exhibited a significant acute drop in IBA1 cell density from DIV0 to DIV1 (Tukey's multiple comparisons one‐way ANOVA, p < 0.05). This initial loss did not persist; by DIV8, microglial numbers stabilized and demonstrated a partial recovery toward baseline levels.

To characterize the astrocyte population and evaluate potential phenotypic shifts during culture, multi‐marker quantification was performed to distinguish between resting (DAPI+/GS+/GFAP‐) and reactive (DAPI+/GFAP+) astrocytes (Figure 3d). Across all conditions, total astrocyte densities closely mirrored those of overall DAPI+ levels, demonstrating a gradual, non‐significant decline from DIV0 through DIV8. The case‐specific astrocyte densities mirrored the microglial trends, with the PSP case (NM4) displaying the highest absolute numbers and the non‐neurological control (NM7) the lowest. The PSP case (NM4) also exhibited a slightly elevated population of reactive astrocytes compared to LBD (NM5) and the non‐neurological control (NM7). Tracking the relative proportions of these subpopulations revealed that the percentage ratio of resting‐to‐reactive astrocytes remained highly stable across the entire culturing period (Figure 3d, right). Strikingly, mechanical slicing at DIV1 did not induce a significant shift toward a reactive phenotype, nor did prolonged cultivation to DIV8 alter the baseline proportional composition substantially within the evaluated cases. Indicating astrocyte populations are less affected by the slicing procedure than the microglial pool in our setup.

The astrocytic numerical stability was mirrored by morphological evaluations of GFAP cells over time. Notably, DIV0 astrocytes displayed uniformly extended processes, whereas DIV1 and, more specifically, DIV8 astrocytes exhibited more heterogeneity in their branches, with fewer long branches displayed. Despite these visual differences, branch count, and total branch length revealed no significant increases (Figure S3) (Tukey's multiple comparisons, one‐way Anova for GFAP count, two way Anova for branch‐count and ‐length, p>0.05), confirming that the cultivation process did not evoke structural astrogliosis [48, 49]. In contrast, both branch number and total branch length declined progressively over time, relative to earlier DIVs.

### 3.3 Standard Bulk Assessment Reveals Little Change Over DIV While CARS Lipid Analysis Spots Subtle Myelin Changes

Myelin integrity over the culturing process is essential to a model of subtle changes in myelin structure. FluoroMyelin live‐imaging (Figure 4A) shows comparable myelin architecture between DIV1 and DIV7, albeit with an increased background and a reduction in the sharpness of myelin structures at later DIVs. In contrast, label‐free CARS‐imaging (Figure 4b) revealed more pronounced fragmentation and diffuse extracellular background by DIV8, even in the raw CARS images prior to extracting or analysing any lipid spectral data. Low‐magnification overviews captured widespread lipid droplet accumulation across the tissue landscape. Conversely, high‐magnification panels resolved individual axonal profiles, and these fields were targeted away from dense lipid accumulations to avoid laser‐induced plasma damage, though locating these clear regions at DIV8 required extended screening times. Overall, the visual alterations in myelin remained modest, with early‐culture stages providing the lowest baseline background.

**FIGURE 4:**
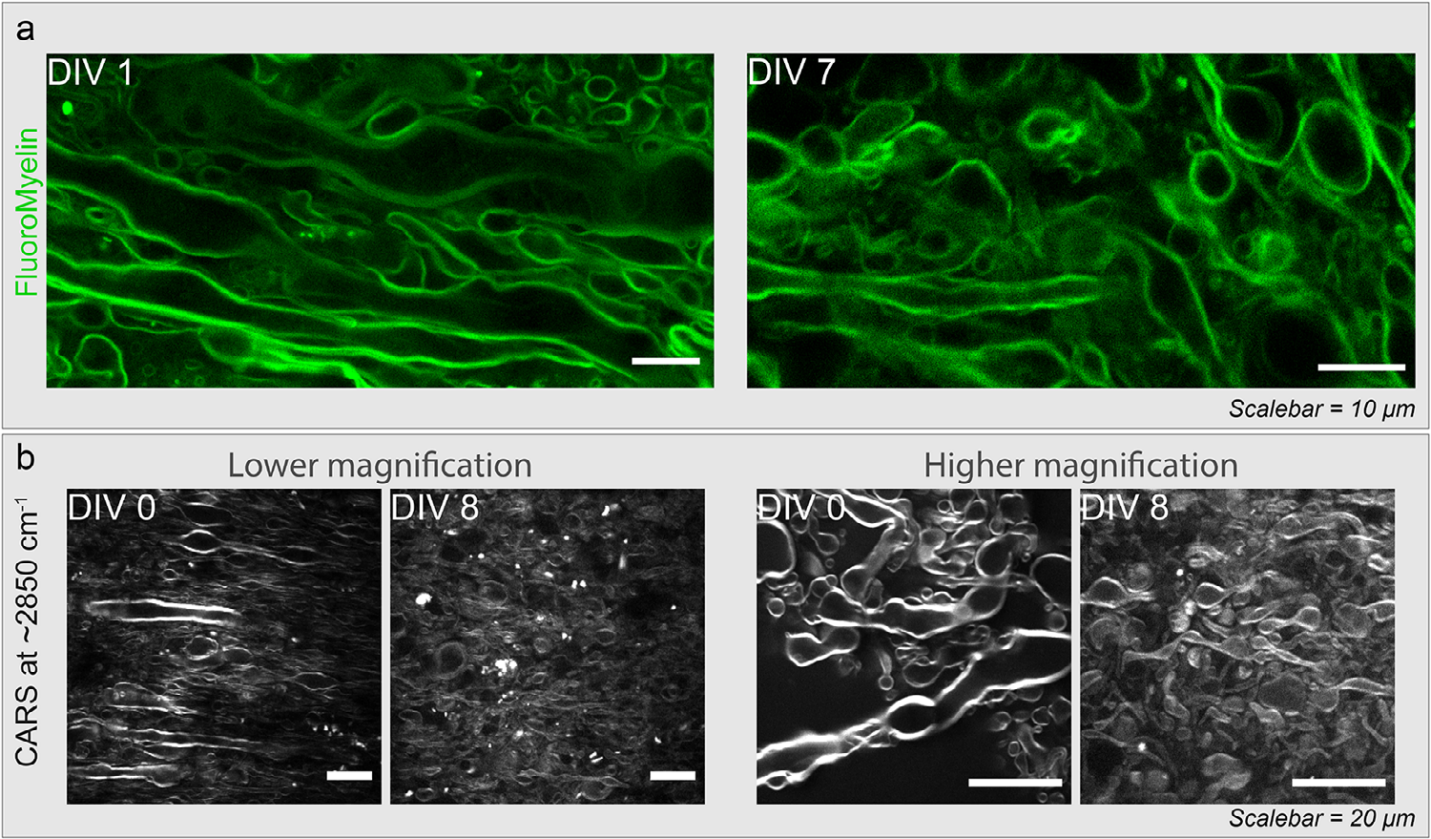
FluoroMyelin and CARS‐imaging, visual highlights in cultured myelin. (a) Confocal live‐cell imaging of 2 mm slices dyed with FluoroMyelin on DIV1 and DIV7 show myelin in green. Scale bars: 10 µm (b) Grey‐scale CARS live‐cell imaging around 2850cm‐1 show myelin and lipid‐droplets in higher‐intensities at this Raman wavenumber due to an abundance in CH2 bonds. DIV0 slices were 200 µm; we cultured DIV8 slices at 2 mm thickness and sliced thinner prior to imaging to reach the approximate slice centre. CARS images at left‐side imaged at a lower magnification than the right side. Scale bars: 20 µm. DIV, days in vitro; CARS, Coherent anti‐Stokes Raman spectroscopy.

Previous studies from our labs in fixed‐frozen human CC reported that myelin swellings in MS donor normal‐appearing WM (NAWM) are characterized by different CH2 bond peaks compared to control tissue [39]. To gain specific information on the lipid‐relevant changes previously reported in MS tissue [39, 50, 51, 52], we limited data acquisition to four Raman wavenumbers, with emphasis on ∼2850 cm^−^^1^, which is the CH_2_ symmetric stretch, indicative of total lipid content and long‐chain fatty acids [53, 54, 55, 56].

Figure 5 illustrates the laser power normalised CARS spectra for DIV0 and DIV8 myelin in non‐MS donor tissue. A marked reduction in the CH_2_ peak at ∼2850 cm^−^^1^ was observed in DIV8 samples relative to DIV0 (with cluster mean peak‐intensity in arbitrary units (a.u.) of 0.80, 0.63, 0.32 averaging 0.58 for DIV0, and 0.48, 0,26, 0.20 averaging 0.31 for DIV8), indicating a loss of lipid‐rich structures. K‐means clustering further revealed that relative cluster differences reduced in DIV8 when compared to DIV0 tissue, while some regions remained relatively unaffected by culturing, broader analysis revealed that spectral profiles typical of DIV0 myelin became less frequent, e.g. distinct initial curve in DIV0 flattening out across wavenumbers in DIV8, and exhibited overall lower peak intensities in DIV8 samples. This result differs from the imaging with FluoroMyelin staining (see Figure 4), underscoring the sensitivity of CARS spectral‐analysis in detecting subtle biochemical alterations, and supporting the powerful combination of this technique with OSCs to reveal otherwise difficult to the detect changes in myelin stability overtime.

**FIGURE 5:**
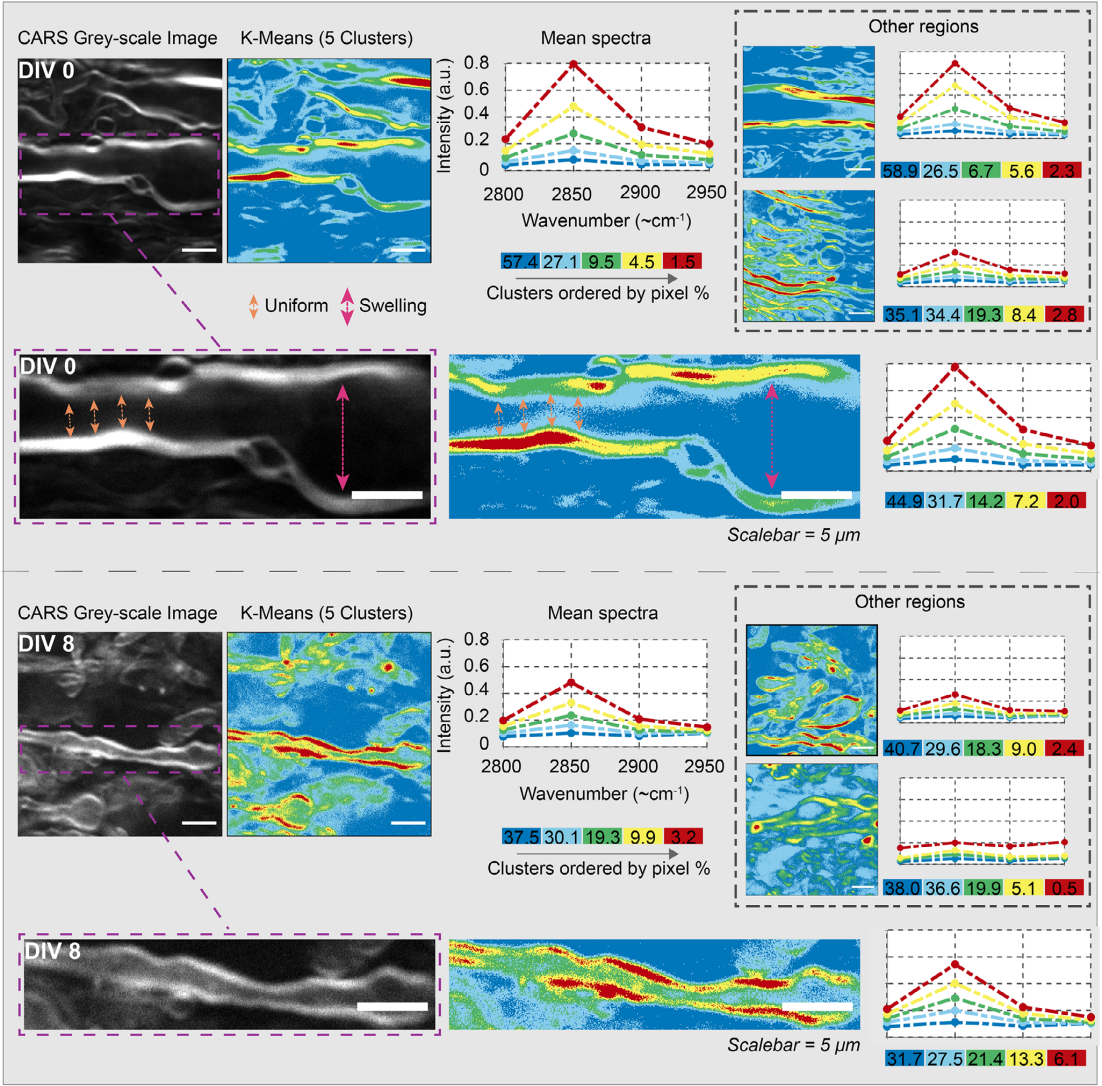
K‐means clustering analysis of spectral data acquired from CARS‐imaging of non‐MS OSCs. Images acquired from two separate non‐MS donors. Scale bars: 5 µm. On the utmost left, Grey‐scale CARS‐images at ∼2850 cm^−1^, followed by the same area's spectral information over 4 different wavenumbers (∼2800, ∼2850, ∼2900, ∼2950) partitioned by k‐means clustering into 5 distinct clusters. Denoted by purple dashed boxes: k‐means clustering on just the cropped region containing a single axon. Orange arrows represent uniform distance between axons, whereas bigger magenta arrows represent swellings; only one image is used as an example, and not all aberrations are marked. Next to the images, a graph displays the mean spectra per cluster; on the y‐axis, the intensity in arbitrary units after normalisation of laser power, and on the x‐axis, the Raman wavenumbers. Below the graphs, data show the percentage of total pixels each cluster occupies. On the right, in black dotted‐line boxes, k‐means cluster analysis of other regions within the slice. DIV0 and DIV8‐data are below and above the dashed line, respectively. DIV0 slices were 200 µm thick; we cultured DIV8 slices at 2 mm thickness and sliced thinner prior to CARS‐imaging to reach the approximate slice‐centre. DIV, days in vitro; CARS, Coherent anti‐Stokes Raman spectroscopy.

To determine whether earlier time points also exhibited myelin alterations, we further analyzed DIV1 tissue, which due to collection and transfer represented a state approximately 32 h after TOD (Figure 6). Overall, no substantial differences between DIV0 and DIV1 myelin were visible (with cluster mean peak‐intensity in a.u. of 0.55, 0.41, 0.28 averaging 0.41, and 0.48, 0.57, 0.40 averaging 0.48), suggesting that the alterations observed at DIV8 arise during later stages of the culturing process. Notably, tissues employed for Figures 5 and 6 come from non‐MS and MS donors, respectively. Closer comparison reveals, how even prior to culturing, at DIV0, MS myelin exhibits modest but consistent reduction in peak intensities (with cluster mean peak‐intensity in a.u. averaging 0.58 for Non‐MS, and averaging 0.41 for MS) and a spatial redistribution of k‐means red‐cluster signals within axonal regions, e.g. highest CH_2_ peaks noted in areas of swollen myelin in contrast to non‐MS. Thus, suggesting pre‐existing biochemical differences and confirming our previous observations on NAWM fixed tissue from MS donors [39]. These findings indicate that CARS‐imaging can detect both culture‐induced and disease‐related changes in myelin composition with high specificity.

**FIGURE 6:**
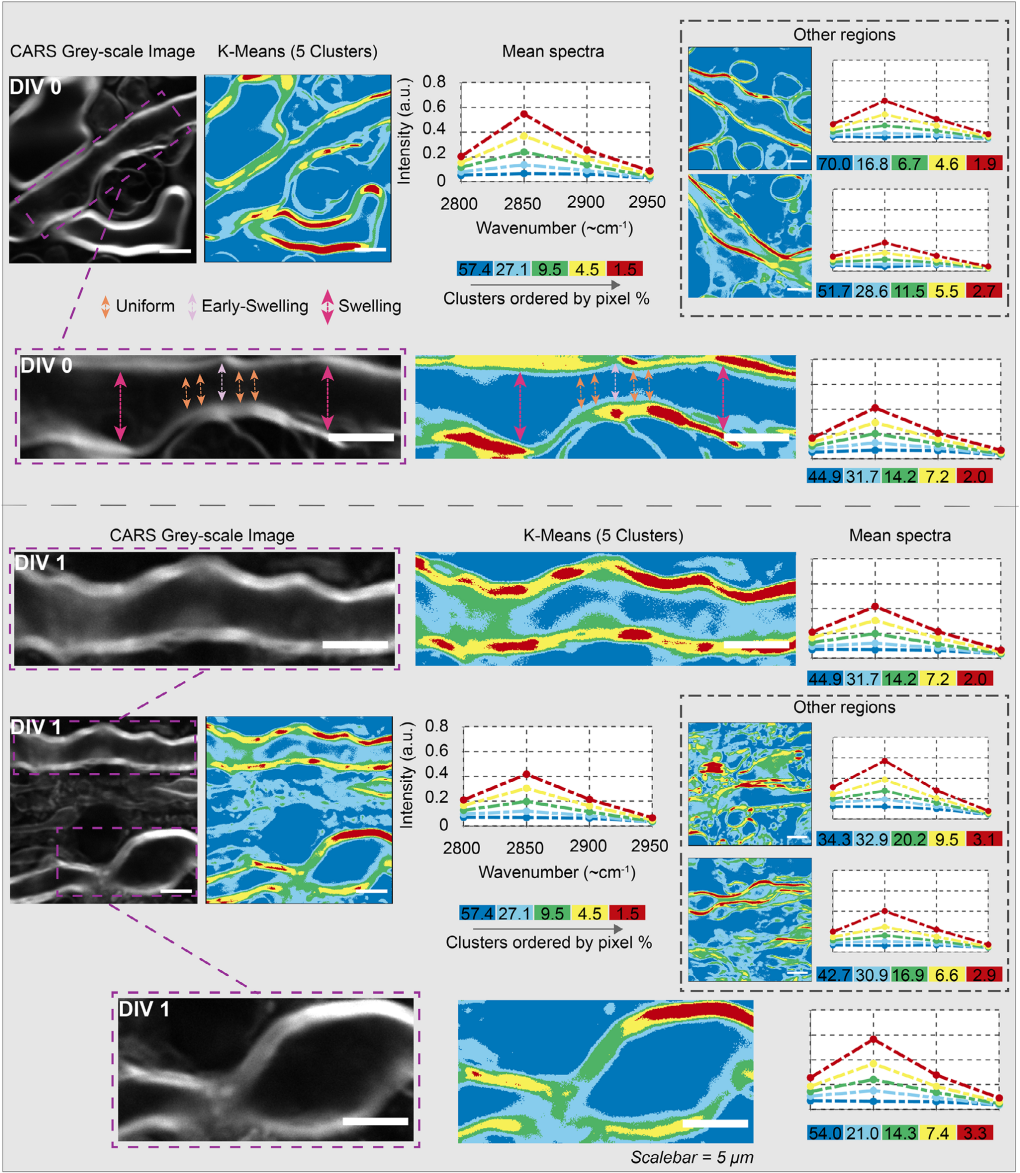
K‐means clustering analysis of spectral data acquired from CARS‐imaging of MS OSCs. Images acquired from one MS donor, but with separate slices. Scale bars: 5 µm. On the utmost left, Grey‐scale CARS‐images at ∼2850 cm^−1^, followed by the same area's spectral information over 4 different wavenumbers (∼2800, ∼2850, ∼2900, ∼2950) partitioned by k‐means clustering into 5 distinct clusters. Denoted by purple dashed boxes: k‐means clustering on just the cropped region containing a single axon. Orange arrows represent uniform distance between axons, whereas bigger pink and magenta arrows represent early swellings and swelling, respectively. Only one image is used as an example; not all aberrations are marked. Next to the images, a graph displays the mean spectra per cluster; on the y‐axis, the intensity in arbitrary units after normalisation of laser power, and on the x‐axis the Raman wavenumbers. Below the graphs, the data show what percentage of total pixels each cluster occupies. All images are of 200 µm thick slices; DIV0 and DIV1‐data are below and above the dashed line, respectively. DIV, days in vitro; CARS, Coherent anti‐Stokes Raman spectroscopy.

## 4 Discussion

Research on the prodromal stages of demyelination in diseases like MS has lingered behind largely due to a lack of accurate tools and the absence of directly translatable animal models [1]. In this study, we have demonstrated the power of CARS microscopy for high‐resolution, label‐free visualization of myelin‐rich CC tissue. To extend the dynamic study period and maintain in vivo‐like myelin morphology and physiology, we have established a novel culture medium enabling tissue viability for at least 8 DIV, confirmed by LDH and confocal imaging, and preserved near‐baseline myelin structure for 32 h post‐mortem, as assessed by CARS microscopy. Furthermore, the combination of CARS‐imaging and *k*‐means analysis provides a robust method for analysing myelin abnormalities in living post‐mortem human brain slices. This represents, to our knowledge, the first application of CARS microscopy to a dynamic living human‐brain‐slice culture, providing a unique platform for studies of myelin‐related diseases like MS, for which no suitable animal model exists.

For culturing, we relied on an air‐liquid interface approach [4] more recently also utilised with OSCs from human post‐mortem material, albeit in cortical tissue [31]. We initially employed an adapted artificial CSF medium [15], but sought to create a novel, chemically defined medium adjusted to our specific needs. In our choices, we limited variability and kept concentrations close to physiological concentrations. From our base medium, we chose to omit HEPES, phenol red, and L‐glutamine. We deemed HEPES inclusion unnecessary [14, 57] due to the presence of a sodium bicarbonate system and the constant use of incubators. We omitted phenol red to minimize possible spectral interference, and adjusted L‐alanyl‐L‐glutamine [58], Mg^2+^ and Na^+^ [59] to better approximate physiological levels in human CSF. As the slicing disrupts energy delivery through the vasculature system, we compensated by elevating D‐Glucose concentrations above physiological levels [60, 61, 62] in line with others using human OSCs [15, 18]. Another highlighted feature of our medium is our serum‐free approach; aimed to minimize variability known to arise from batch‐to‐batch differences, avoid introducing unknown components, and work toward supporting animal‐free research, the latter in line with global efforts of the FDA and EMA to promote alternatives to animal testing [2, 3]. We also sought to avoid additional sources of reactive astrogliosis; a reported effect initiated by the inclusion of serum [63]. We thus generated a near‐physiological base medium less affected by uncertain media composition, considering it most suited to allow for robust comparisons between experimental groups.

Thinner slices of 0.45 and 1.4 mm showed enhanced viability when compared to 2 mm‐thick specimens, while recognising that thicker slices provide advantages for flash freezing purposes or when requiring a higher degree of the original 3D‐architecture. Notably, we acquired the 1.4 and 2 mm slices using a slice‐matrix, providing a cost‐ and time‐effective alternative to vibratome sectioning. While viability remained promising, by DIV8, CARS imaging revealed a diminished myelin signal in most axons, specifically CH_2_, and an increased lipid body accumulation. Consequently, the higher density of CH_2_ heavy molecules within lipid bodies increased the risk of plasma‐related photodamage [64], thereby limiting suitable regions as lipid bodies had to be avoided in the field of view during high‐magnification CARS‐imaging. While higher magnification scans provide a work‐around, navigating around these dense lipid‐accumulations dramatically increased the time required to screen for viable imaging fields at DIV8. Overall, our results suggest that early‐culture imaging can be helpful in minimizing artefacts, increasing the quality of myelin visualization, and reducing the presence of lipid bodies in the tissue.

Another goal was to delineate a window most suitable for observing undeterred myelin, for example to allow for application of pharmacological agents relevant to myelin degeneration in MS in future studies. Our viability studies and CARS‐imaging suggest that the first few days post‐mortem, on the border of what is considered acute (i.e. within 24 h) versus long‐term, are useful timeframes to appreciate a still preserved myelin morphology. This timeline is substantially shorter than OSC strategies that culture for 1–2 weeks to reach a stable environment, before moving to further experiments, doing so with the intent of limiting effects of processes such as reactive microglial activation and astrogliosis [65]. In mice OSCs, mature tissue tends to be less viable and take longer to stabilize than the typical 3–5 days required for younger tissue [66, 67, 68]. Conversely, our LDH data reveal a different pattern: while early‐stage cell loss does occur, it is not excessive, and stabilization sets in quickly; comparable, for example, to prior research showing resected human cortical slices stabilizing at the end of DIV1 [17]. Moreover, after a few DIV in a previous study, post‐mortem tissue neurons appeared more viable and displayed a lower incidence of reactive glial cells even when compared to resected tissue directly [26]. Overall, our data and previous studies employing similar OSCs time‐windows [17, 69, 70] underscore the usefulness of a short‐term OSC approach.

MHC‐II and IBA1 profiling revealed case‐dependent microglial dynamics that resolved acute, injury‐induced activation without triggering chronic inflammation. While NNC and PSP tissues maintained population stability, LBD tissue exhibited an acute microglial decline at DIV 1 before recovering by DIV 8. Because non‐microglial lineages like oligodendrocyte precursor cells can transiently express MHC‐II under stress [71], evaluating MHC‐II alongside the pan‐microglial marker IBA1 provided critical cellular context. Cross‐referencing these population dynamics highlighted a striking proportional shift: while DIV1 IBA1 cell counts were either stable (PSP) or significantly reduced (LBD), both neurodegenerative cases upregulated MHC‐II at DIV 1. This rapid mobilization into an active state indicated that pre‐existing neurodegeneration primed the microglial pool, rendering it highly reactive to mechanical trauma, a phenomenon established in the literature regarding disease‐associated microglial priming [72]. Ultimately, while slicing acted as the initial catalyst, the kinetics and magnitude of this neuroinflammatory response were governed by the baseline disease state.

In contrast, the astrocyte population demonstrated remarkable phenotypic and structural stability. Total and case‐specific astrocyte densities mirrored microglial trends, highest in PSP and lowest in NNC, reinforcing that baseline pathology dictated the overall glial landscape. Crucially, the ratio of resting‐to‐reactive astrocytes remained unchanged by slicing or extended cultivation. Morphologically, a progressive decline in branch number and length reflected subtle environmental adaptation rather than hypertrophic remodeling. These findings suggest that microglia served as the primary sensors of mechanical stress, while astrocytes prioritized homeostatic stability, preventing a progressive, multi‐lineage inflammatory cascade. Collectively, the resolution of acute microglial reactivity without chronic gliosis underscores the stability of this platform, demonstrating that human OSCs effectively preserved the unique traits dictated by each donor's specific disease background.

Analysis of the CARS spectral information revealed myelin alterations in cultured tissue, which were undetectable by FluoroMyelin staining. This could mean that with traditional staining, prolonged culture studies may inadvertently analyse already‐altered myelin, with uncertain degrees of variation, potentially leading to a false sense of normality, an uncertainty CARS‐imaging can remove. While we have offered a suggested window of analysis, the absence of CARS data between DIV1 and DIV8 could warrant further investigation into the exact onset of pronounced myelin alterations. The data suggest these changes are a slow process, as some DIV8 myelin still exhibited spectral signatures comparable to DIV0. Beyond culture effects, we characterized the specific decrease in CH_2_ bonds in MS myelin, relative to non‐MS, consistent with CARS data on swellings previously published by our group [39], using *k*‐means cluster analysis to distinguish and accentuate molecular differences within axonal myelin. Swellings and myelin blisters can occur in both MS and non‐MS tissue, but are more frequently observed in MS [39, 40], suggesting that focused analysis may yield important insights into the disease pathology. Placing the relevance within a larger biological context, a reduction in CH_2_ peaks aligns with evidence of diffuse lipid alterations and increased lipid polarity in MS NAWM [52, 73], which can compromise myelin insulation and decrease action potential conduction velocity [73]. This altered composition may render the myelin sheath more permeable to extracellular ions, inducing axonal ion leakage and altered excitability [41]. This mechanism is particularly relevant given recent findings connecting aberrant axonal activity directly to the instigation of degenerative myelin swellings [74]. At this stage, it remains to be disentangled whether these CH_2_ alterations initiate this pathological cascade, occur in response to primary axonal physiology changes that disrupt axo‐myelinic communication [41], or arise from a primary immune attack against the myelin sheath. Our present work focused on baseline and end‐point comparisons; future studies incorporating dynamic CARS‐imaging of OSCs could shed light on the temporal progression of myelin abnormalities. For instance, there is still a need to understand the dynamics behind de‐ and remyelination in MS brains, and analyzing the effects of de‐ and re‐myelination in human tissue has shown suitability in CC‐tissue in similar animal model OSC studies [75]. Emerging evidence demonstrates that changes in axonal physiology interact with myelin lipid biochemical alterations to exacerbate subtle myelin aberrations, such as swellings [74]. Dynamic CARS monitoring of myelin biochemistry over time will help clarify this link between axonal activity and downstream demyelination cascades. Crucially, the flexibility of the readout in live human tissue enables human‐context drug screening to identify compounds that can halt this structural degradation, providing solid mechanistic conclusions and new therapeutic strategies.

## 5 Conclusions

We have provided characterization of the development of a human myelin model within which we have delineated a window of sustained viability and myelin properties. We have combined this with the use of non‐linear optics methods (CARS) capable of providing high‐quality information on the subtle changes of myelin structure and composition; of relevance in studying otherwise difficult‐to‐detect changes in prodromal phases of demyelination in diseases like MS.

## Author Contributions

Everyone was involved in reviewing and editing. In addition, KRK: Data curation, conceptualization, formal analysis, investigation, methodology, project administration, software, validation, visualization, and original draft preparation. JPK: Conceptualization, methodology, resources, and software. FT: Methodology and investigation. GJS: Conceptualization, funding acquisition, and resources. HLO: Conceptualization, funding acquisition, methodology, project administration, resources, software, and supervision. AL: Conceptualization, funding acquisition, methodology, project administration, resources, and supervision.

## Funding

AL and HLO obtained funding for this study from the Dutch National MS Foundation. Grant Number: 2021‐008. AL obtained funding from the National Multiple Sclerosis Society, Progressive MS Alliance Award Grant Number: PA 2021–36033. AL and GJS obtained funding from Stichting MS Research, alternative for animal‐free research, Grant Number: MS24‐1223.

## Conflicts of Interest

The authors declare no conflict of interest.

## Supporting information




**Supporting File**: advs76471‐sup‐0001‐SuppMat.docx

## Data Availability

The data that support the findings of this study are available from the corresponding author upon reasonable request.
