# Supplementary material for: Label‐Free Myelin Fingerprinting: An Adult Human Post‐Mortem Brain Slice Platform via Coherent Anti‐Stokes Raman Spectroscopy
Source: Adv Sci (Weinh). 2026 Jul 11:e76471. Online ahead of print. doi: 10.1002/advs.76471 (PMC13355935; doi:10.1002/advs.76471)
Supplement: Supplementary file 1 — Supporting File: advs76471‐sup‐0001‐SuppMat.docx [file ADVS-9999-e76471-s001.docx]

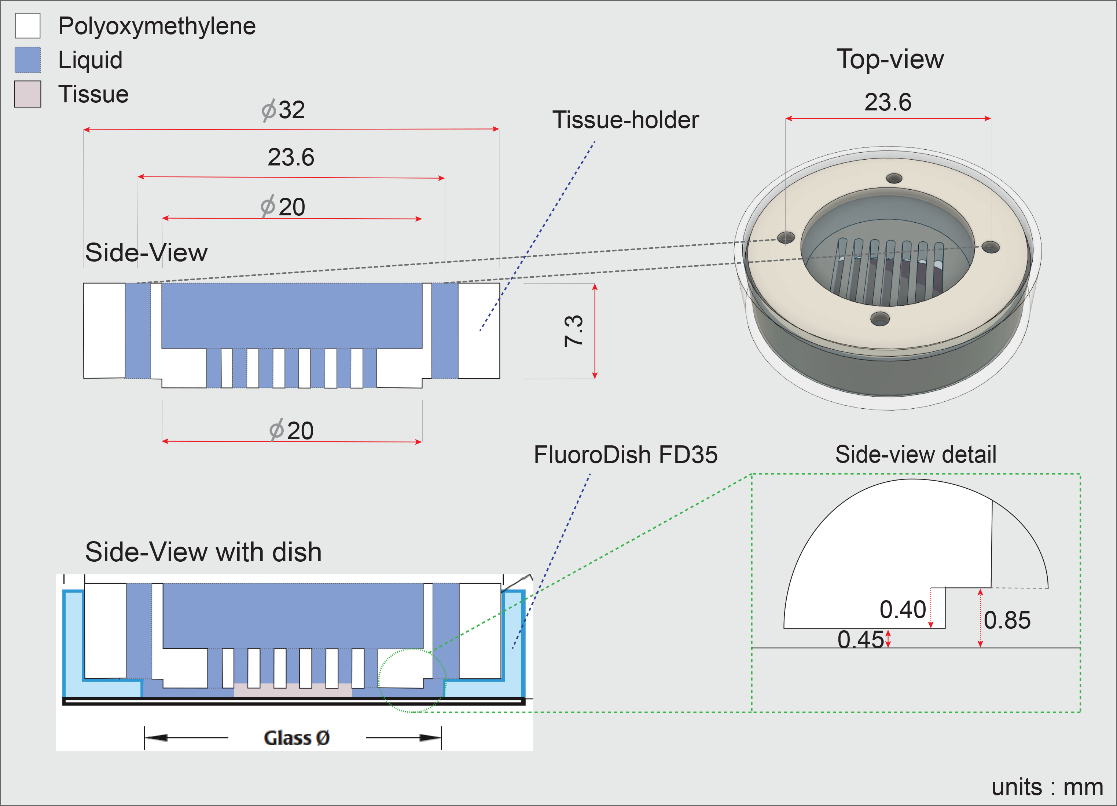
Supporting information

**Fig S1. Sketch for custom-made tissue holder** Legend showing the tissue holder’s material is polyoxymethylene in white, liquid such as culture medium that can flow through certain parts of the tissue holder in darker blue, and the tissue in pink held down by the tissue holder to reduce mechanical drift during imaging. The tissue holder is designed to fit in the FluoroDish FD35 and imaging is done through the dish’s glass imaging-window at the bottom. These specifics, in mm, are adjusted for tissue of 500micron thick. The tissue holder leaves 450 of space between the Fluorodish glass and the tissue-holder, which means there 50 micron is pushed down on the tissue. For varying sizes the space between FluoroDish glass and tissue-holder can be adjusted by altering dimensions. Holder is cleaned with an appropriate alcohol solution and re-used.


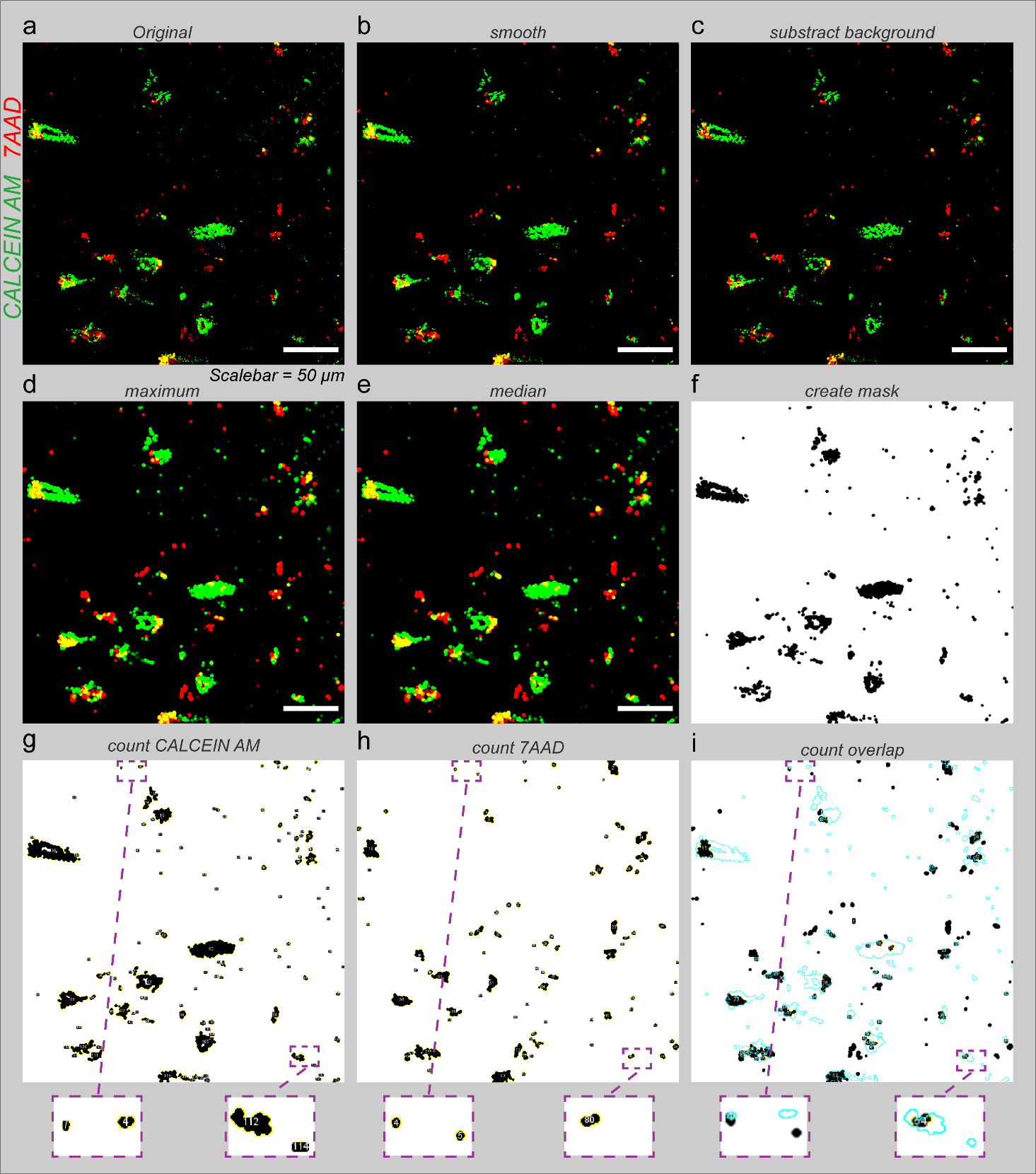


**Fig S2. Post-processing steps of Calcein AM and 7AAD cell-counts.** Example of a confocal image of dual (calcein AM & 7-AAD) stained slice region: green, red, yellow denote live, dead, and live with a damaged cell membrane, respectively. Images have the same brightness and contrast settings for fair comparisons. Scale bar: 50μm. (a) Original image converted to 8-bit (b-c) Smoothing and background subtraction for clean-up purposes. (d-e) Due to the punctate signal, a single cell may give rise to multiple smaller signals. By using the maximum and median functions we enlarge signals, so they’ll overlap if in close proximity, to count as a single event in later steps, reducing double positives. (f) Turns data into binary values and creates a mask, in the example only the mask for the Calcein AM channel is shown. (g-h) Shows an automatic count of cells displaying Calcein AM and 7AAD staining respectively. (i) An overlap of the previous two mask shows which cells are live but damaged; this is a manual counting process due to the occurrence of double positives. We adjusted final live, dead, and live but damaged numbers accordingly. Calcein AM, calcein acetoxymethyl; 7AAD, 7-amino-actinomycine D.


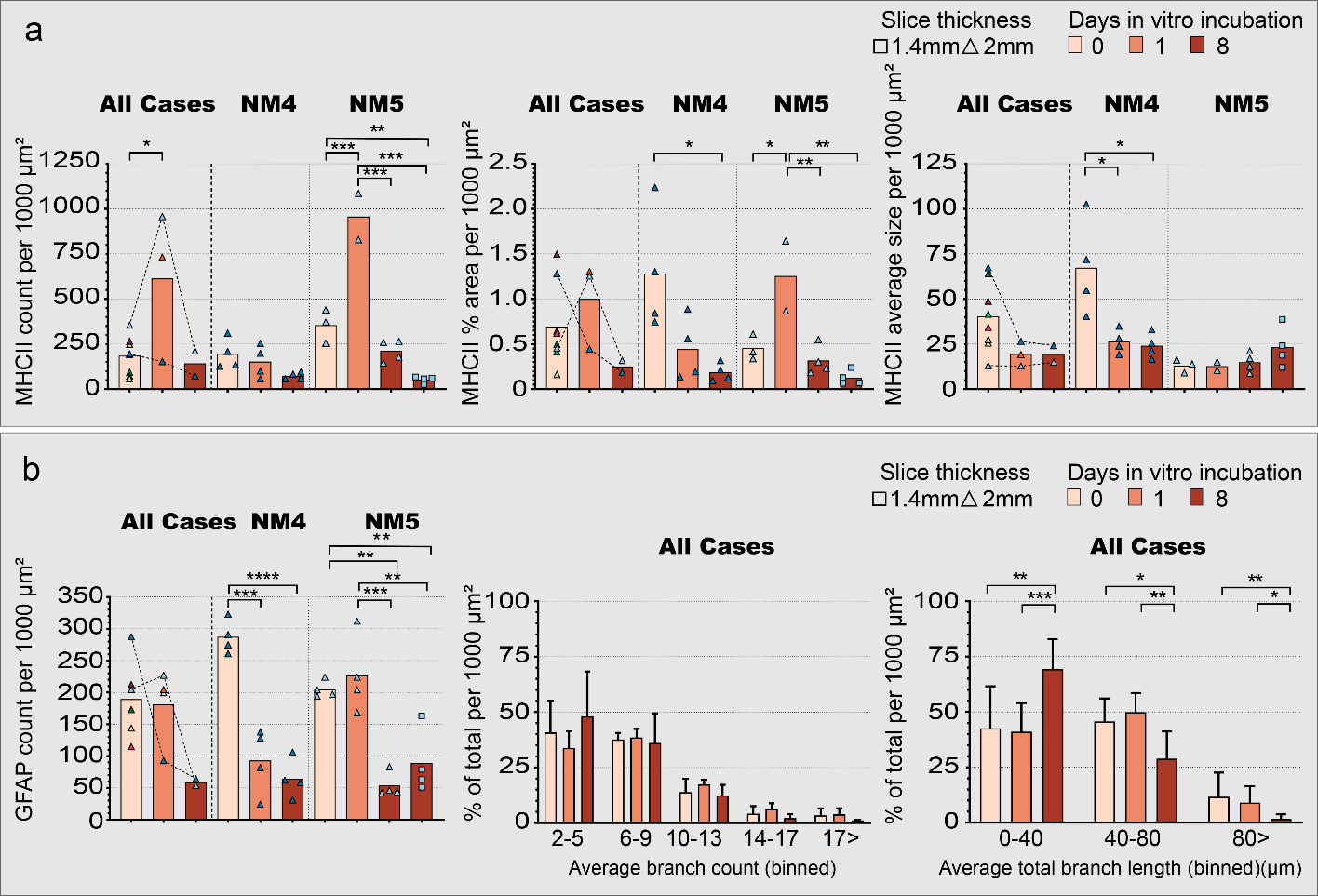
**Fig S3. IHC-staining of MHCII and GFAP in OSCs.** For all graph-symbols: squares and triangles denote slices 1.4 and 2mm thick, respectively. Error bars show standard deviation. (a-b) “All cases” graph-groups display counts on DIV0 (n=8), DIV1 (n=3), DIV8 (n=2) (n=donor-mean, each data-point is an average of 2-4 slice-regions within a singular slice). Matching colours and connecting lines denote data from the same donor. NM4 and NM5 display slice means (n=region within a singular slice). (b) Three graphs from left to right showing, MHCII particle count, %area and average size over an area of 1000µm2. (c) Three graphs from left to right showing, GFAP count, and binned average branch counts and total branch lengths over an area of 1000µm2. Tukey’s multiple comparisons one-way Anova was performed for most statistical tests, except GFAP branch count and length which were two-way Anova’s (* p < 0.05, ** p < 0.005, *** p < 0.0005). GFAP, glial fibrillary acidic protein; MHCII, major histocompatibility complex class II; DIV, days in vitro

Code S4

% HYPERSPECTRAL K-MEANS CLUSTERING (PUBLICATION VERSION)

clc;

clear;

close all;

warning off;

% COLOR PALETTE

base_colors = [ **...**

**0.000** **0.467** **0.730**; **...** % Blue

**0.530** **0.800** **0.930**; **...** % Light Blue

**0.310** **0.700** **0.400**; **...** % Green

**0.969** **0.941** **0.337**; **...** % Yellow

**0.730** **0.000** **0.067**; **...** % Dark Red

**0.850** **0.325** **0.098**; **...** % Orange

**0.667** **0.267** **0.600**; **...** % Purple

**0.466** **0.674** **0.188**; **...** % Lime

**0.933** **0.200** **0.467**; **...** % Magenta

**0.635** **0.078** **0.184**; **...** % Maroon

% SELECT INPUT FILES

[tifFile, tifPath] = uigetfile('*.tif', **...**

'Select hyperspectral TIFF');

**if** isequal(tifFile,**0**)

**return**;

**end**

[pFile, pPath] = uigetfile('*.txt', **...**

sprintf('Select power file for %s', tifFile));

**if** isequal(pFile,**0**)

**return**;

**end**

% LOAD POWER FILE

pdata = load(fullfile(pPath, pFile));

% Column definitions

wnumbers = pdata(:,**2**);

wnumbers = wnumbers(:)';

pumppower = pdata(:,**3**);

stokespower = pdata(:,**4**);

power = pumppower.^**2** .* stokespower;

power = power ./ max(power); %power normalization

% READ TIFF STACK

info = imfinfo(fullfile(tifPath, tifFile));

nWaves = length(wnumbers);

fprintf('TIFF pages: %d\n', length(info));

fprintf('Power entries: %d\n', nWaves);

**if** length(info) ~= nWaves

error('Mismatch: TIFF pages (%d) != wavenumbers (%d)', **...**

length(info), nWaves);

**end**

img = zeros(info(**1**).Height, info(**1**).Width, nWaves);

fprintf('Loading hyperspectral stack...\n');

**for** i = **1**:nWaves

A = imread(fullfile(tifPath, tifFile), i, 'Info', info);

img(:,:,i) = A ./ power(i);

**end**

% BACKGROUND CORRECTION + NORMALIZATION

img = img - **199**;

img = mat2gray(img, [**0** **4095**]);

% PREVIEW MEAN IMAGE

meanImg = mean(img,**3**,'omitnan');

figure('Name','Mean Image');

imshow(meanImg,[]);

title('Mean Intensity Image');

% USER INPUT: NUMBER OF CLUSTERS

answer = inputdlg('Number of clusters (k):', **...**

'K-means clustering', **1**, {'3'});

**if** isempty(answer)

**return**;

**end**

k = str2double(answer{**1**});

**if** isnan(k) || k < **2**

error('k must be integer >= 2');

**end**

**if** k > size(base_colors,**1**)

error('Maximum supported clusters = %d', size(base_colors,**1**));

**end**

% USER INPUT: CLUSTER ORDERING

orderChoice = questdlg( **...**

'How should clusters be ordered?', **...**

'Cluster ordering', **...**

'Mean Intensity', **...**

'Pixel Count (%)', **...**

'Mean Intensity');

**if** isempty(orderChoice)

**return**;

**end**

% PREPARE DATA FOR CLUSTERING

[nRows, nCols, nBands] = size(img);

data = reshape(img, [], nBands);

% K-MEANS CLUSTERING

fprintf('Running k-means (k = %d)...\n', k);

rng(**42**);

[idx, ~] = kmeans(data, k, **...**

'Replicates', **3**, **...**

'MaxIter', **500**);

% CLUSTER ORDERING

**if** strcmp(orderChoice, 'Pixel Count (%)')

counts = histcounts(idx, **1**:k+**1**);

[~, order] = sort(counts, 'descend');

**else**

clusterMeans = zeros(**1**,k);

**for** c = **1**:k

clusterMeans(c) = mean(data(idx==c,:), 'all');

**end**

[~, order] = sort(clusterMeans, 'ascend');

**end**

new_idx = zeros(size(idx));

**for** c = **1**:k

new_idx(idx == order(c)) = c;

**end**

idx = new_idx;

% RESHAPE TO IMAGE SPACE

clusterMap = reshape(idx, nRows, nCols);

colors = base_colors(**1**:k,:);

% COMPUTE MEAN SPECTRA + PERCENTAGES

meanSpectra = zeros(k, nBands);

percentages = zeros(**1**, k);

**for** c = **1**:k

mask = (idx == c);

percentages(c) = **100** * sum(mask) / numel(idx);

**if** any(mask)

meanSpectra(c,:) = mean(data(mask,:), **1**);

**end**

**end**

% BUILD RGB CLUSTER IMAGE

clusterRGB = zeros(nRows, nCols, **3**);

**for** c = **1**:k

mask = (clusterMap == c);

**for** ch = **1**:**3**

tmp = clusterRGB(:,:,ch);

tmp(mask) = colors(c,ch);

clusterRGB(:,:,ch) = tmp;

**end**

**end**

% VISUALIZATION

figure('Name','K-means Results','Position',[**100** **100** **1200** **500**]);

subplot(**1**,**2**,**1**)

imshow(clusterRGB)

title(sprintf('Cluster Map (k = %d)', k))

subplot(**1**,**2**,**2**)

hold on

**for** c = **1**:k

plot(wnumbers, meanSpectra(c,:), **...**

'Color', colors(c,:), **...**

'LineWidth', **2**);

**end**

xlabel('Wavenumber')

ylabel('Intensity')

title('Mean Cluster Spectra')

grid on

xlim([min(wnumbers) max(wnumbers)])

xticks(wnumbers)

legendText = cell(k,**1**);

**for** c = **1**:k

legendText{c} = sprintf('C%d (%.1f%%)', c, percentages(c));

**end**

legend(legendText, 'Location', 'best');

% CONSOLE OUTPUT

fprintf('\nCluster percentages:\n');

fprintf('--------------------\n');

**for** c = **1**:k

fprintf('Cluster %d: %.2f %%\n', c, percentages(c));

**end**
